# Adaptive recursive algorithm for optimal weighted suprathreshold stochastic resonance

**DOI:** 10.1098/rsos.160889

**Published:** 2017-09-13

**Authors:** Liyan Xu, Fabing Duan, Xiao Gao, Derek Abbott, Mark D. McDonnell

**Affiliations:** 1 Institute of Complexity Science, Qingdao University, Qingdao 266071, People's Republic of China; 2 Computational and Theoretical Neuroscience Laboratory, Institute for Telecommunications Research, School of Information Technology and Mathematical Sciences, University of South Australia, Adelaide, South Australia 5095, Australia; 3 Centre for Biomedical Engineering (CBME) and School of Electrical & Electronic Engineering, The University of Adelaide, Adelaide, South Australia 5005, Australia

**Keywords:** suprathreshold stochastic resonance, adaptive signal processing, Kalman–least mean square, recursive algorithm

## Abstract

Suprathreshold stochastic resonance (SSR) is a distinct form of stochastic resonance, which occurs in multilevel parallel threshold arrays with no requirements on signal strength. In the generic SSR model, an optimal weighted decoding scheme shows its superiority in minimizing the mean square error (MSE). In this study, we extend the proposed optimal weighted decoding scheme to more general input characteristics by combining a Kalman filter and a least mean square (LMS) recursive algorithm, wherein the weighted coefficients can be adaptively adjusted so as to minimize the MSE without complete knowledge of input statistics. We demonstrate that the optimal weighted decoding scheme based on the Kalman–LMS recursive algorithm is able to robustly decode the outputs from the system in which SSR is observed, even for complex situations where the signal and noise vary over time.

## 1. Introduction

Stochastic resonance in multi-threshold systems was initially investigated in [1], where the input signal is subthreshold. More interestingly, the concept of suprathreshold stochastic resonance (SSR) was also introduced in multi-threshold systems [2–4]. Note that SSR is an important variation of stochastic resonance [5,6], where the output is counterintuitively enhanced by noise, operating with signals of arbitrary magnitude, not restricted to weak or subthreshold signals. Since its introduction, SSR has received considerable attention in diverse areas concerned with the transmission of signals, and has also been considered in the design of cochlear implants [7], analogue-to-digital converter circuits [8–10], nonlinear detectors [11,12], digital accelerometers [13] and stochastic quantizers [14]. In the seminal works of Stocks and co-workers [2–4], the nonlinearity in each element of the model is assumed to be a binary quantizer. Thus, such threshold systems can be described as stochastic signal quantizers that have been analysed in terms of lossy source coding and quantization theory [14–16]. Following the works in [14–16], we investigated the decoding scheme of quantized signal, named as optimal weighted decoding, by using weighted coefficients [17,18]. Our results show that under certain conditions the performance of the optimally weighted quantizer response is superior to that of the original unweighted arrays [17,18].

However, the previous studies [17,18] have been undertaken assuming that the input characteristics are statistically stationary, and the decoding schemes are based on *a priori* knowledge of the inputs. Specifically, the noise is modelled as white Gaussian distributed [17,18]. However, in practical applications, the statistical characteristics of the input signals are generally unknown or are often varied with time. Furthermore, noise in real systems is coloured, and the idealization of white noise is never exactly realized [19,5]. These constraints severely limit the decoding operation based on performance enhancement of real systems. A highly successful solution to this more difficult problem is found in adaptive filtering, which is a powerful approach with a wide variety of engineering applications [20–24]. Adaptive filtering has the ability to adjust system parameters automatically with no *a priori* knowledge of inputs, and allows processing the case wherein the properties of inputs are unknown, non-stationary or time variable [25–27]. Interestingly, a toy model has been established that illustrates a process of optimization that works without *a priori* knowledge of the input statistical distribution—so we do have a precedent to show mathematical tractability of this class of problem [28].

Specifically, the Kalman filter and the least mean square (LMS) algorithm are two of the most popular adaptive estimation methods in adaptive signal processing, with the former as a realization of the optimal Bayesian estimator and the latter as a recursive solution to the optimal Wiener filtering problem [27]. Recently, Mandic *et al.* subtly developed a joint perspective on these two algorithms, and proposed the Kalman–LMS recursive algorithm that permits the implementation of Kalman filters without any notion of Bayesian statistics [29].

The purpose of this paper is to extend optimal-weighted decoding to more general input characteristics by using the Kalman–LMS recursive algorithm, wherein the weighting coefficients can be adaptively adjusted based on real-time measurements of the input signals. For the typical SSR model of threshold arrays, we find this Kalman–LMS recursive algorithm can deal with not only the simple situation of stationary signals, but also more complicated cases of non-stationary signals and coloured noise. The decoding performance of the mean square error (MSE) distortion obtained by the Kalman–LMS recursive algorithm illustrates interesting progress in optimal weighted SSR that may be of benefit in adaptive signal processing algorithms applied to nonlinear noisy systems.

## 2. Model and method

### 2.1. Model

**Figure 1.**
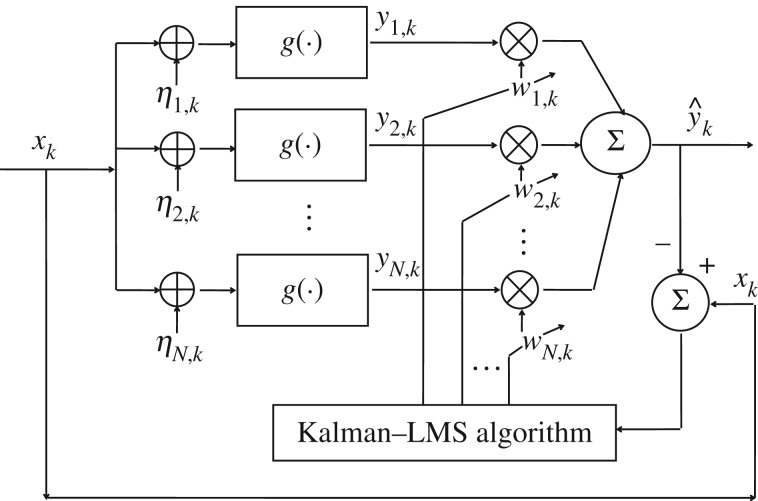
Adaptively weighted summing array of *N* noisy nonlinear elements, *g*(⋅). Each element operates on a common signal *x*_*k*_ subject to additive noise *η*_*i*,*k*_ at time *k*. The output of each individual element *y*_*i*,*k*_ is multiplied by the weighting coefficient *w*_*i*,*k*_, and the overall output ${\hat{y}}_{k}=\sum_{i=1}^{N}w_{i,k}y_{i,k}$.

We consider an adaptively weighted summing array of *N* noisy nonlinear elements, as shown in figure 1. All elements receive the same input signal *x*_*k*_ representing the *k*th element in the time series, which is assumed to be a deterministic signal or a stationary stochastic process [29]. Each element of the array is endowed with the same input–output characteristic, modelled by the static (memoryless) function g. The *i*-th nonlinear element is subject to independent and identically distributed (i.i.d.) additive noise component *η*_*i*,*k*_ with standard deviation *σ*_*η*_, which is independent of the signal *x*_*k*_. Accordingly, each element produces the output signal *y*_*i*,*k*_=g[*x*_*k*_+*η*_*i*,*k*_]. The output signal *y*_*i*,*k*_ is multiplied by the weighting coefficient *w*_*i*,*k*_ (*w*_*i*,*k*_∈ℜ). *w*_*i*,*k*_ are adjusted by the Kalman–LMS recursive algorithm aiming to minimize the performance metric of MSE. All weighted outputs are summed to give the overall output of array ${\hat{y}}_{k}=\sum_{i=1}^{N}w_{i,k}\;y_{i,k}$.

The Kalman–LMS recursive algorithm combines Kalman filtering with LMS-type algorithms to control both the direction and magnitude of adaptation steps along the shortest path so as to achieve the global minimum of MSE [29]. Our purpose is to adaptively adjust the weights by this algorithm to make the decoding output ${\hat{y}}_{k}$ approximate the input *x*_*k*_, i.e. minimizing the MSE distortion.

### 2.2. Method

To begin, we introduce a column vector **y**_*k*_=[*y*_1,*k*_,*y*_2,*k*_,…,*y*_*N*,*k*_]^⊤^ to represent the *N* output signals of nonlinear elements for each random input *x*_*k*_. We denote a vector of weights as **w**_*k*_=[*w*_1,*k*_,*w*_2,*k*_,…,*w*_*N*,*k*_]^⊤^ and the optimal weights as ${\text{w}}_{k}^{o}$ that corresponds to the minimum of MSE.

#### 2.2.1. Performance evaluation criteria

Ideally, we wish to achieve

2.1 $$x_{k}={\text{y}}_{k}^{⊤}{\text{w}}_{k}^{o},$$

where the aim is to estimate the optimal weight vector ${\text{w}}_{k}^{o}$ for minimizing the MSE distortion. It can be fixed, i.e. ${\text{w}}_{k}^{o}={\text{w}}^{o}$, or time varying as in equation (2.1) [29]. For the stationary input signal *x*_*k*_ and noise *η*_*k*_, equation (2.1) can be written as:

2.2 $$x_{k}={\text{y}}_{k}^{⊤}{\text{w}}^{o}.$$

When the inverse matrix ${\left({\text{y}}_{k}{\text{y}}_{k}^{T}\right)}^{-1}$ exists, the Wiener optimal weight vector **w**^o^ is given by ${\text{w}}^{o}={\left({\text{y}}_{k}{\text{y}}_{k}^{T}\right)}^{-1}{\text{y}}_{k}x_{k}$ [16,21]. For the stationary input *x*_*k*_, the memoryless nonlinearity g and the stationary noise *η*_*k*_, we assume the optimal weight vector **w**^*o*^_*k*_ converges to the Wiener solution of **w**^o^. We desire to estimate the optimal weight vector **w**^o^ recursively, based on the existing weight vector **w**_*k*−1_, the input signal *x*_*k*_ and output signals of nonlinear elements **y**_*k*_, i.e. ${\hat{\text{w}}}^{o}={\text{w}}_{k}=f({\text{w}}_{k-1},x_{k},{\text{y}}_{k})$. Then the decoding output is ${\hat{y}}_{k}={\text{y}}_{k}^{⊤}{\text{w}}_{k-1}$. The error between the input *x*_*k*_, and the decoding output ${\hat{y}}_{k}$ is given by

2.3 $$e_{k}=x_{k}-{\hat{y}}_{k}=x_{k}-{\text{y}}_{k}^{⊤}{\text{w}}_{k-1}.$$

Similarly, the weight error vector ${\tilde{\text{w}}}_{k}$ between the weight vector estimate **w**_*k*_ and the optimal weight vector **w**^o^ is written by [29]

2.4 $${\tilde{\text{w}}}_{k}={\text{w}}^{o}-{\text{w}}_{k}$$

and its contribution to the error *e*_*k*_ is given by

2.5 $$e_{k}={\text{y}}_{k}^{⊤}{\tilde{\text{w}}}_{k-1}.$$

The decoding performance metric of MSE represents the power of the output error *e*_*k*_, and is expressed as

2.6 $$\mathrm{MSE}=E[e_{k}^{2}],$$

where the statistical expectation *E*(⋅) introduced in equation (2.6) is defined in terms of the joint probability of the output **y**_*k*_ and the weight error vector ${\tilde{\text{w}}}_{k}$. The theoretical calculation of MSE is difficult, and we will numerically obtain the MSE distortion for a sufficiently large observation time in the following experiments.

For the memoryless function g, we assume the output **y**_*k*_ at the recursion step *k* is not related to the weight error vector ${\tilde{\text{w}}}_{k-1}$ at the time step *k*−1. Thus, the instantaneous time-varying MSE is given in [29]

2.7 $$\varsigma_{k}=E[{\left({\text{y}}_{k}^{⊤}{\tilde{\text{w}}}_{k-1}\right)}^{2}]={\text{y}}_{k}^{⊤}{\text{P}}_{k-1}{\text{y}}_{k},$$

where ${\text{P}}_{k-1}=E[{\tilde{\text{w}}}_{k-1}{\tilde{\text{w}}}_{k-1}^{⊤}]$ is the symmetric and positive semi-definite weight error covariance matrix, and the statistical expectation *E*(⋅) is calculated in terms of the probability density of ${\tilde{\text{w}}}_{k-1}$. In [29], another performance evaluating metric—the mean square deviation *J*_*k*_—is introduced, which represents the power of the weight error vector ${\tilde{\text{w}}}_{k}$ and is given by

2.8 $$J_{k}=E[{\tilde{\text{w}}}_{k}^{⊤}{\tilde{\text{w}}}_{k}]=\mathrm{tr}({\text{P}}_{k}).$$

The mean square deviation *J*_*k*_ is related to the instantaneous MSE in equation (2.7) through the weight error covariance matrix **P**_*k*_. Therefore, minimizing *J*_*k*_ is equivalent to minimizing the instantaneous MSE [29]. Based on equation (2.8), we will deduce the weight vector estimate **w**_*k*_ at each recursion step *k*.

#### 2.2.2. Optimal learning gain

The LMS algorithm uses the stochastic gradient descent and uses a recursive estimation of the optimal weight vector, **w**^o^ in equation (2.2), in the form ${\text{w}}_{k}={\text{w}}_{k-1}-\mu_{k}\nabla_{\text{w}}E[e_{k}^{2}]$, where *μ*_*k*_ is a step size and ∇_**w**_ is a gradient vector [29]. Based on the instantaneous estimate $E[e_{k}^{2}]\approx e_{k}^{2}$, the LMS solution is given in [21]

2.9 $${\text{w}}_{k}={\text{w}}_{k-1}+\mu_{k}{\text{y}}_{k}e_{k}.$$

Note that the second term in equation (2.9) the weight update, *μ*_*k*_**y**_*k*_*e*_*k*_, has the same direction as the vector **y**_*k*_. It turns out that the gradient descent performs locally optimal steps but has no means to follow the globally optimal shortest path to the solution **w**^o^. Therefore, it is necessary to control both the direction and magnitude of the adaptive steps *μ*_*k*_ to follow the shortest, optimal path to **w**^o^ [29]. In this way, Mandic *et al.* introduce a positive definite learning gain matrix **G**_*k*_, in the context of Kalman filters, to replace the scalar step size *μ*_*k*_ so as to control both the direction and magnitude of the gradient descent [29]. Thus, the weight update recursion in equation (2.9) generalizes to

2.10 $${\text{w}}_{k}={\text{w}}_{k-1}+{\text{G}}_{k}{\text{y}}_{k}e_{k}={\text{w}}_{k-1}+{\text{g}}_{k}e_{k},$$

where the gain vector **g**_*k*_=**G**_*k*_**y**_*k*_ [29].

Subtracting **w**^o^ from both sides of equation (2.10) and replacing the error with equation (2.5), we can rewrite equation (2.10) in terms of the weight error vector as [29]

2.11 $${\tilde{\text{w}}}_{k}={\tilde{\text{w}}}_{k-1}-{\text{g}}_{k}{\text{y}}_{k}^{⊤}{\tilde{\text{w}}}_{k-1}.$$

Utilizing equation (2.11), we can obtain the recursion for the weight error covariance matrix **P**_*k*_ as [29]

2.12 $${\text{P}}_{k}=E[{\tilde{\text{w}}}_{k}{\tilde{\text{w}}}_{k}^{⊤}]={\text{P}}_{k-1}-({\text{P}}_{k-1}{\text{y}}_{k}{\text{g}}_{k}^{⊤}+{\text{g}}_{k}{\text{y}}_{k}^{⊤}{\text{P}}_{k-1})+{\text{g}}_{k}{\text{g}}_{k}^{⊤}{\text{y}}_{k}^{⊤}{\text{P}}_{k-1}{\text{y}}_{k}.$$

Substituting equation (2.12) into equation (2.8), the mean square deviation is obtained as [29]

2.13 $$J_{k}=J_{k-1}-2{\text{g}}_{k}^{⊤}{\text{P}}_{k-1}{\text{y}}_{k}+∥{\text{g}}_{k}{∥}^{2}{\text{y}}_{k}^{⊤}{\text{P}}_{k-1}{\text{y}}_{k}.$$

This derivation of equation (2.13) uses the facts that $\mathrm{tr}({\text{P}}_{k-1}{\text{y}}_{k}{\text{g}}_{k}^{⊤})=\mathrm{tr}({\text{g}}_{k}{\text{y}}_{k}^{⊤}{\text{P}}_{k-1})={\text{g}}_{k}^{⊤}{\text{P}}_{k-1}{\text{y}}_{k}$ and $\mathrm{tr}({\text{g}}_{k}{\text{g}}_{k}^{⊤})={\text{g}}_{k}^{⊤}{\text{g}}_{k}=∥{\text{g}}_{k}{∥}^{2}$.

Based on equation (2.13), the optimal learning gain vector **g**_*k*_ can be obtained by differentiating *J*_*k*_ with respect to **g**_*k*_, setting to zero and solving for **g**_*k*_, to give [29]

2.14 $${\text{g}}_{k}=\frac{{\text{P}}_{k-1}{\text{y}}_{k}}{{\text{y}}_{k}^{⊤}{\text{P}}_{k-1}{\text{y}}_{k}}.$$

This optimal gain vector is precisely the Kalman gain [22]. Substituting equation (2.14) into equation (2.12), the update for **P**_*k*_ is then obtained

2.15 $${\text{P}}_{k}={\text{P}}_{k-1}-{\text{g}}_{k}{\text{y}}_{k}^{⊤}{\text{P}}_{k-1}.$$

#### 2.2.3. Kalman–LMS recursive algorithm

From equations (2.10), (2.14) and (2.15), Kalman–LMS recursive algorithm of estimating the optimal weights **w**^o^ is outlined as:

At each instant *k*>0, based on measurements (*x*_*k*_,**y**_*k*_)

(1) compute the optimal learning gain (Kalman gain):
  $${\text{g}}_{k}=\frac{{\text{P}}_{k-1}{\text{y}}_{k}}{\left({\text{y}}_{k}^{⊤}{\text{P}}_{k-1}{\text{y}}_{k}+\delta \right)},$$
  where the constant *δ*>0 is an initial disturbance for preventing from recursion stop. Without loss of generality, the symmetric and positive semi-definite matrix **P**_0_ is chosen as unit matrix.
(2) update the weight vector estimate:
  $${\text{w}}_{k}={\text{w}}_{k-1}+{\text{g}}_{k}(x_{k}-{\text{y}}_{k}^{⊤}{\text{w}}_{k-1}).$$
(3) update the weight error covariance matrix:
  $${\text{P}}_{k}={\text{P}}_{k-1}-{\text{g}}_{k}{\text{y}}_{k}^{⊤}{\text{P}}_{k-1}.$$

The above operating process exhibits that Kalman–LMS recursive algorithm iteratively updates the weights after each sample, which requires little or no *a priori* knowledge of the signal or noise characteristics. A proof of convergence of the mean square deviation for stationary inputs is provided in appendix A. In the meanwhile, the fastest convergence time corresponding to the array of *N* static nonlinear elements in figure 1 is also analysed. Our analysis shows that the fastest convergence time is about *N* times sampling time Δ*t*. It means that, for a given sampling time Δ*t*, the larger the parallel array size *N* is, the longer the convergent time is. This argument will be shown in the following experiments.

## 3. Results

It is interesting to note that the above-mentioned decoding scheme using the Kalman–LMS recursive algorithm can be applied to an array composed of arbitrary nonlinear elements. Here, we consider the static function g of figure 1 as Heaviside function that is a typical threshold element [2,3]. The individual output *y*_*i*,*k*_ is given by the response function

3.1 $$y_{i,k}=\left\{ \begin{array}{ll} 1 & x_{k}+\eta_{i,k}>\theta_{i},\\ 0 & \text{otherwise,} \end{array}\right.$$

where *θ*_*i*_ (*i*=1,2,…,*N*) is the threshold level for each Heaviside function g.

In the following, we will explore two cases of input characteristics, i.e. stationary and non-stationary, to examine the MSE distortion performance of optimal weighted decoding scheme based on the Kalman–LMS recursive algorithm. In addition, for the noise components *η*_*i*,*k*_ in figure 1, white Gaussian and coloured noises are considered, respectively.

### 3.1. Gaussian noise with identical thresholds

We first consider the case where all threshold levels in equation (3.1) are identical, i.e. *θ*_*i*_=*θ*, and the noises *η*_*i*,*k*_ are Gaussian distributed.

It is well known that the stationary assumption of inputs is ideal and is inadequate for dealing with situations in which non-stationarity of the signal and or noise is intrinsic to the problem. The ability to adapt in a non-stationary environment is an important function of an adaptive algorithm. Specifically in a non-stationary environment it can offer a tracking capability for the time-varying input signal, provided that the variations are sufficiently slow [24].

**Figure 2.**
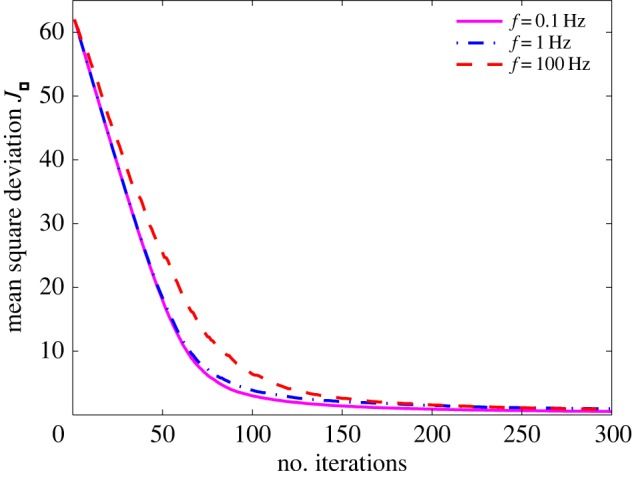
Comparison of mean square deviation for non-stationary inputs with various time variations. The non-stationary input is Gaussian distributed with standard deviation $\sigma_{x}(t)=\sin(2\pi ft)$ (*f*=0.1 Hz, 1 Hz and 100 Hz). Here, the array size *N*=63, the noise standard deviation *σ*_*η*_=1 and sampling time Δ*t*=10^−3^ s.

We now consider the case where the input *x*_*k*_ is non-stationary stochastic signal. For instance, *x*_*k*_ is Gaussian distributed, but the standard deviation *σ*_*x*_(*t*) is time varying. Here, the signal standard deviation is chosen as $\sigma_{x}(t)=\sqrt{2}\sin(2\pi ft)$ with the modulation frequency *f*, and the sampling time Δ*t*=10^−3^ s. Of course, we can have other forms of *σ*_*x*_(*t*) provided that it is slow time-varying. In appendix A, we investigate the tracking capability of the proposed Kalman–LMS algorithm, and an intrinsic time scale of *N*Δ*t* is approximately provided for analysing the temporal dynamics of the adaptive process. For different time variations of the non-stationary input and the array size *N*=63, a performance comparison is shown in figure 2. It apparently shows that, when the modulation frequency *f*=0.1 Hz and 1 Hz, the mean square deviation *J*_*k*_ can track non-stationary changes of the environment well, since such time variations of standard deviation *σ*_*x*_(*t*), compared with the intrinsic time scale of *N*Δ*t*, occur slowly enough.

Unless specifically mentioned, all results in the following parts are obtained for the example case where the input signal for non-stationary characteristics is Gaussian distributed with standard deviation $\sigma_{x}(t)=\sqrt{2}\sin(2\pi t)$, and the input signal for stationary characteristics is Gaussian distributed with *σ*_*x*_=1.

**Figure 3.**
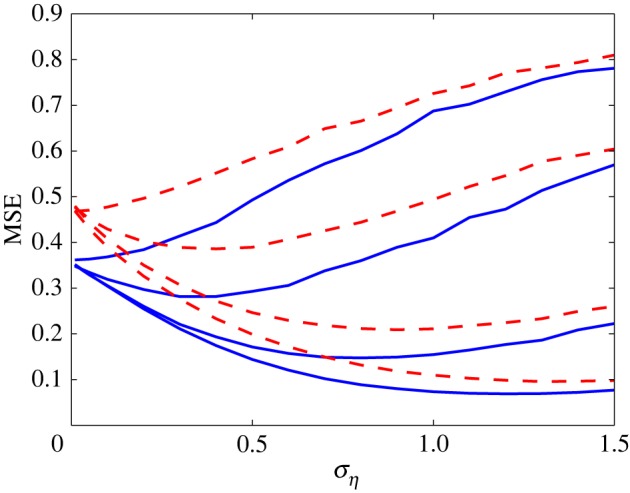
MSE distortion versus *σ*_*η*_ for Gaussian noise with identical thresholds. The dashed red lines represent the MSE distortion for non-stationary input that is Gaussian distributed with standard deviation $\sigma_{x}(t)=\sqrt{2}\sin(2\pi t)$. The solid blue lines correspond to the MSE distortion for stationary input that is Gaussian distributed with *σ*_*x*_=1. For the two cases of input characteristics, from top to bottom the array sizes are *N*=1,3,15 and 63.

The MSE distortions for the non-stationary stochastic input are plotted in figure 3 (dashed red lines). Figure 3 clearly illustrates that the optimal value of *σ*_*η*_ for minimizing the MSE distortion is nonzero for *N*>1, and thus SSR occurs. As *N* increases, the MSE distortion at the optimal *σ*_*η*_ decreases, while the optimal value of *σ*_*η*_ will gradually increase, even to a value larger than unity for *N*=63.

For comparison, the solid blue lines correspond to the MSE distortion curves for stationary Gaussian input signal with standard deviation *σ*_*x*_=1. It is noted that these two inputs have been chosen to give the same average power. For a sufficient time duration, their average powers are all unity. It is clear in figure 3 that the MSE value is far larger for the non-stationary case than stationary case. In addition, for two cases of the input characteristics, very similar qualitative behaviours are all seen. Thus, this also validates that the SSR effect is quite general, not restricted to the stationary signals considered in [17,18].

### 3.2. Coloured noise with identical thresholds

In the above studies, noise is assumed to be white Gaussian distributed. In the physical world, however, such an idealization is never exactly realized. The effects of various noise correlation times on stochastic resonance have been previously investigated [5,19,30–37]. Owing to the practical importance of coloured noise, we next evaluate the optimal weighted decoding performance under the coloured noise circumstances.

We consider the model driven by an additive exponentially correlated Gaussian noise, i.e. Ornstein–Uhlenbeck noise (OU noise). The archetypal source for OU noise is given in [19]

3.2 $$\dot{\xi }(t)=-\frac{1}{\tau }\xi +\frac{\sqrt{D}}{\tau }\xi_{w}(t),$$

where *ξ*_*w*_(*t*) denotes Gaussian white noise with autocorrelation 〈*ξ*_*w*_(*t*)*ξ*_*w*_(*s*)〉=2*δ*(*t*−*s*) and *D* is the noise intensity. The stationary autocorrelation of *ξ*(*t*) with correlation time *τ* is then represented by [19]

3.3 $$\langle \xi (t)\xi (s)\rangle =\left(\frac{D}{\tau }\right)\exp\left(-\frac{|t-s|}{\tau }\right).$$

When correlation time τ→0, equation (3.3) reproduces the white-noise source frequently used in stochastic resonance studies.

**Figure 4.**
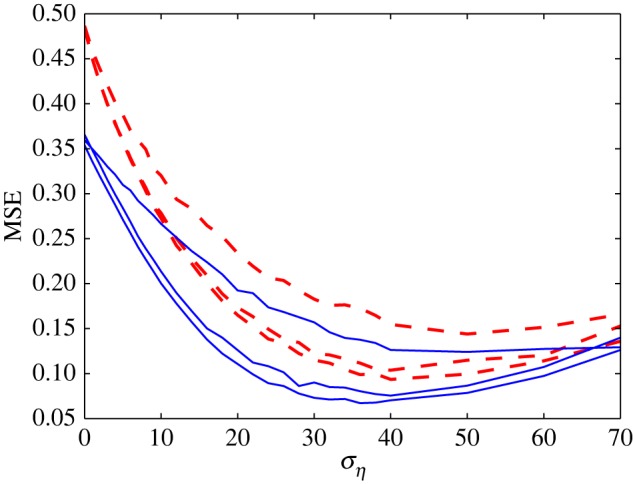
MSE distortion versus standard deviation *σ*_*η*_ for coloured noise with different values of correlation time *τ*. The dashed red lines represent the non-stationary input, which is Gaussian distributed with time-varying standard deviation $\sigma_{x}(t)=\sqrt{2}\sin(2\pi t)$. The solid blue lines correspond to the stationary input, which is Gaussian distributed with *σ*_*x*_=1. For the two cases of input characteristics, from bottom to top *τ*=0.1 s, 1 s and 10 s. Here, the array size *N*=63.

Figure 4 shows the MSE distortion against the standard deviation *σ*_*η*_ of the OU noise with different correlation time *τ*=0.1 s, 1 s and 10 s. Here, the array size is *N*=63. The dashed red lines represent the MSE distortion performance for non-stationary inputs, while the solid blue lines correspond to the decoding performance for stationary inputs. As the correlation time *τ* increases, it is seen in figure 4 that the minimum MSE value at the optimal *σ*_*η*_ increases. The characteristic behaviour for stochastic resonance is in good agreement with results in [5,19,30–32]. The reason is that, since the variance $\sigma_{\eta }^{2}$ of the OU noise is *D*/*τ* in equation (3.3), the noise strength *D* increases proportionally with the increase of *τ* for a given $\sigma_{\eta }^{2}$. However, we assume the output **y**_*k*_ at the recursion step *k* is not related to the weight error vector ${\tilde{\text{w}}}_{k-1}$ at the time step *k*−1 in equation (2.7). This restriction will hinder the application of this adaptive algorithm to the case of coloured noise. Despite this, this adaptive algorithm still plays, and presents the corresponding MSE distortions in figure 4. It is seen that, as the correlation time *τ* increases, the corresponding MSE distortions increase for both the non-stationary (dashed lines) and stationary (solid lines) inputs.

**Figure 5.**
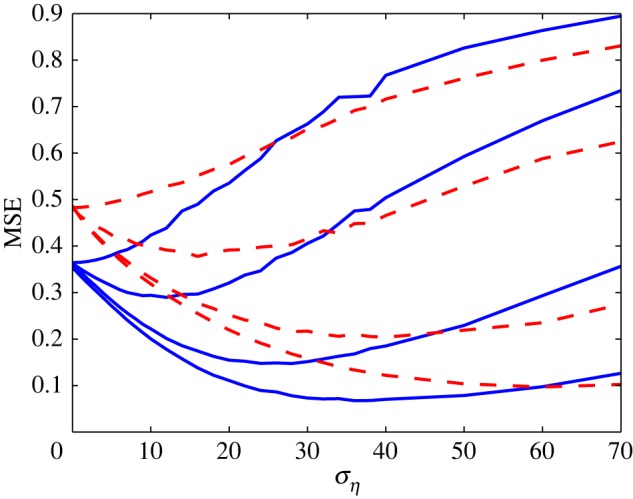
The MSE distortion versus *σ*_*η*_ for coloured noise with the correlation time *τ*=0.1 s in the case of identical thresholds. The solid blue lines are for the case of stationary input, and the dashed red lines correspond to the case of non-stationary input. From top to bottom, the array sizes *N*=1, 3, 15 and 63.

Under coloured noise conditions, to compare the MSE distortion performance for stationary and non-stationary input situations, figure 5 shows MSE for OU noise with the correlation time *τ*=0.1 s and the array sizes *N*=1, 3, 15 and 63.

Note from figure 5 that, as *N* increases, very similar decoding performance appears when compared with figure 3. Specifically, these two figures both indicate that the MSE is far larger for the non-stationary case than in the stationary case. The results in figure 5 show that this Kalman–LMS recursive algorithm can deal with not only the simple situation of stationary signals, but also more complicated cases of non-stationary signal buried in coloured noise.

### 3.3. Gaussian noise with grouped thresholds

Our recent work [18] showed that the decoding performance for multigroup parameter settings is superior to that of identical parameter settings. Following the approach presented in [18], we next study the decoding performance of the Kalman–LMS recursive algorithm for the case of grouped thresholds.

We divide the set of threshold elements into *M* (*M*≤*N*) groups. Within each group, the threshold element size is equal, i.e. *N*/*M*, and the threshold levels are equally spaced and set as *θ*_*m*_=*mσ*_*x*_/(*M*+1) for *m*=1,2,…,*M*.

**Figure 6.**
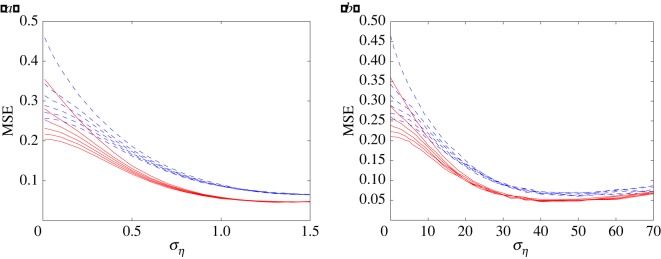
MSE distortion versus the noise level for group sizes *M*=1, 2, 3, 5, 10 and 120 (from top to bottom), and the array size *N*=120. Within each group, the threshold element size is equal, i.e. *N*/*M*, and the threshold levels are equally spaced and set as *θ*_*m*_=*mσ*_*x*_/(*M*+1) for *m*=1,2,…,*M* (*M*≤*N*). (*a*) White Gaussian noise. (*b*) Coloured noise with the correlation time *τ*=0.1 s. The solid red lines correspond to the MSE distortion for stationary input, and the dashed blue lines represent the MSE distortion for non-stationary input.

Figure 6*a* exhibits the MSE distortion for Gaussian noise with various group sizes *M* in the cases of non-stationary and stationary inputs. Here, the array size *N*=120, and the group sizes *M*=1, 2, 3, 5, 10 and 120. It is apparently illustrated in figure 6*a* that, for small noise levels, the decoding performance greatly improves as the group size *M* increases. While, for very large noise levels, all of the MSE values that correspond to different groups tend to the same observation, which equals the MSE distortion of the identical threshold level setting. This fact tells us that, for weak and moderate noise intensities, the multigroup setting scheme can reduce the MSE distortion. Figure 6*a* also reveals that the grouped threshold setting can be extended to adaptively weighted summing arrays.

### 3.4. Coloured noise with grouped thresholds

It is interesting to study the performance of weighted decoding for the case of grouped thresholds under the coloured noise circumstances. Similarly, we also illustrate the MSE curves for OU noise with various group sizes *M* in the cases of non-stationary and stationary inputs in figure 6*b*. It can be seen from figure 6*a*,*b* that, as the group sizes *M* increase, the grouped decoding performance under the coloured noise circumstances is similar to that under Gaussian noise circumstances.

## 4. Conclusion and discussion

**Figure 7.**
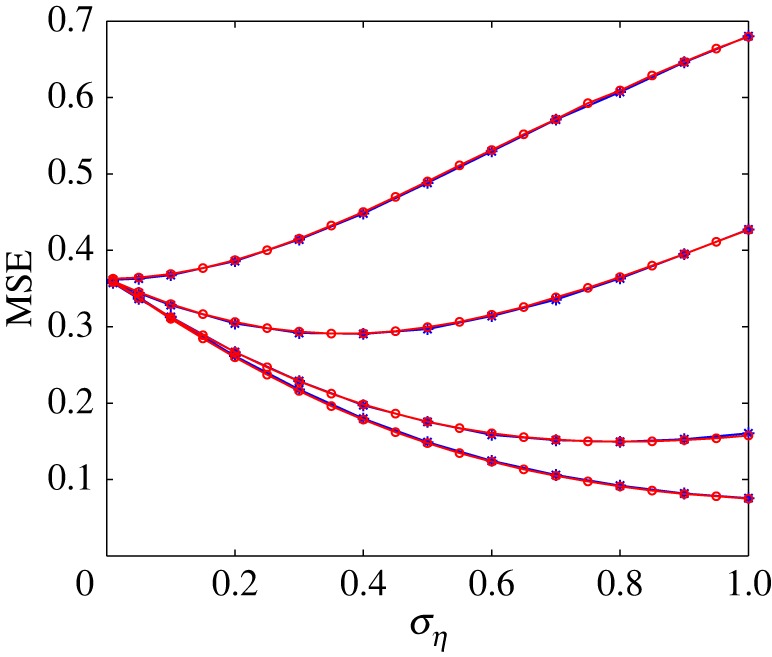
MSE distortion versus the noise level *σ*_*η*_ of Gaussian white noise in the case of identical thresholds. Here, the array sizes *N*=1, 3, 15 and 63 (from top to bottom), and the stationary input signal is Gaussian distributed. The circled red lines correspond to the MSE distortion for Kalman–LMS recursive algorithm, and the blue stars represent the MSE distortion for optimal weighted decoding presented in [17].

In this paper, we extend the optimal weighted decoding approach to more general input characteristics in the model of a weighted summing array of *N* noisy nonlinear elements based on the Kalman–LMS recursive algorithm. A proof of convergence of the mean square deviation for stationary inputs is derived. We especially apply the algorithm to a parallel array of threshold elements and investigate the decoding performance for inputs with stationary, non-stationary characteristics under Gaussian noise and coloured noise circumstances. In the case of stationary inputs, after successive iterations of the Kalman–LMS recursive algorithm it converges to the optimum Wiener solution in some statistical sense [24]. Our previous work [17] has shown that, for the case of identical thresholds, the optimal weighted decoding is equivalent to Wiener linear decoding. Therefore, for stationary inputs, the decoding performance exploiting Kalman–LMS recursive algorithm is consistent with that of optimal weighted decoding in the case of identical thresholds. Figure 7 clearly illustrates that the results for these two methods are the same, and MSE distortion curves completely overlap.

Notably, although for the case of stationary inputs, the decoding performance of these two methods is equal under the condition of identical thresholds, the Kalman–LMS recursive algorithm is simple and generally easy to implement since the output weights are updated following each input signal sample, instead of calculating the output weights using all the training data in one shot. Therefore, compared with the optimal weighted decoding scheme [17], this Kalman–LMS recursive algorithm is a practical method for finding close approximate solutions to equation (2.10) in real time. Moreover, this Kalman–LMS recursive algorithm can adaptively adjust the weighting coefficients so as to minimize the MSE distortion without complete knowledge of input statistics and thus can be applicable to the non-stationary inputs and the coloured noise situations as shown in figures 2 and 3–6 (dashed lines). Additionally, we also apply the Kalman–LMS recursive algorithm to grouped threshold setting for minimizing MSE distortion. The obtained results show that the multigroup setting scheme can be extended to adaptively weighted summing array, and is a significant scheme for many potential applications inspired by SSR mechanism. Although the Kalman–LMS recursive algorithm is applied in the summed array of *N* noisy nonlinear elements, we expect that our findings may be important for future work on more complex models that will stimulate further studies on SSR.

Beyond the memoryless nonlinearity considered in figure 1, we argue that this Kalman–LMS adaptive algorithm may potentially be extended to other nonlinear systems, for instance, the Hodgkin–Huxley neuron model [5], the reduced FitzHugh–Nagumo neuron model [4,32,34], the auditory model [6,7,16] or biomedical devices [38]. However, the dynamical nonlinear system has its intrinsic time scale that controls the evolution of the system state. The system outputs at adjacent time steps are also correlated with each other to a certain extent, even the input signal or the input noise are independently identically distributed. These factors will lead to larger misalignment between the true and estimated weights. Thus, a more general Kalman–LMS adaptive algorithm for estimating the time-varying weight vector needs to be developed.

## Data accessibility

Figures input files: Figshare doi:10.6084/m9.figshare.4214229; Figshare doi:10.6084/m9.figshare.4214232; Figshare doi:10.6084/m9.figshare.4214244; Figshare doi:10.6084/m9.figshare.4214253; Figshare doi:10.6084/m9.figshare.4214262; Figshare doi:10.6084/m9.figshare.4989803.

## Appendix A

First, we analyse the convergence of the proposed adaptive algorithm. Based on equations (2.4) and (2.8), the convergence of **w**_*k*_ to the optimal weight vector **w**^o^ is equivalent to the convergence of the mean square deviation *J*_*k*_. From equation (2.14), the mean square deviation *J*_*k*_ in equation (2.13) can be written as

A 1 $$\begin{array}{rl} J_{k} & \;=J_{k-1}-{\text{g}}_{k}^{⊤}P_{k-1}{\text{y}}_{k}\\ & \;=J_{k-1}-\frac{{\text{y}}_{k}^{⊤}P_{k-1}^{⊤}P_{k-1}{\text{y}}_{k}}{{\text{y}}_{k}^{⊤}P_{k-1}{\text{y}}_{k}}\\ & \;=J_{k-1}-\frac{∥P_{k-1}{\text{y}}_{k}{∥}^{2}}{\varsigma_{k}}. \end{array}$$

Note that $J_{k}=E[∥{\tilde{\text{w}}}_{k}{∥}^{2}]\geq 0$ and ${\text{y}}_{k}^{⊤}P_{k-1}^{⊤}P_{k-1}{\text{y}}_{k}=∥P_{k-1}{\text{y}}_{k}{∥}^{2}\geq 0$, thus we find

A 2 $$0\leq J_{k}\leq J_{k-1},$$

for *ς*_*k*_≠0 and any recursion *k*. In practice, we update the learning gain as

A 3 $${\text{g}}_{k}=\frac{P_{k-1}{\text{y}}_{k}}{{\text{y}}_{k}^{⊤}P_{k-1}{\text{y}}_{k}+\delta }=\frac{P_{k-1}{\text{y}}_{k}}{\varsigma_{k}+\delta }$$

and the mean square deviation *J*_*k*_ becomes

A 4 $$J_{k}=J_{k-1}-\frac{∥P_{k-1}{\text{y}}_{k}{∥}^{2}}{\varsigma_{k}+\delta },$$

for *δ*>0 and *ς*_*k*_+*δ*>0. The inequality of equation (A.2) still holds. Therefore, as k→∞, the mean square deviation $J_{k}\to 0$ is convergent.

Next, we discuss the convergence time of the adaptive algorithm. In order to obtain the fastest convergence time, we need to maximize the term of ∥*P*_*k*−1_**y**_*k*_∥^2^/*ς*_*k*_ in equation (A.1). Since *P*_*k*−1_ is a symmetric positive semi-definite matrix, thus we have the normal form

A 5 $$P_{k-1}=Q\Lambda_{k-1}Q^{⊤},$$

with the normalized orthonormal matrix *QQ*^⊤^=*I* and the eigenvalue matrix *Λ*_*k*−1_=diag[λ_1_,λ_2_,…,λ_*N*_]. Then, we can rewrite the term

A 6 $$\frac{{\text{y}}_{k}^{⊤}P_{k-1}^{⊤}P_{k-1}{\text{y}}_{k}}{{\text{y}}_{k}^{⊤}P_{k-1}{\text{y}}_{k}}=\frac{{\text{y}}_{k}^{⊤}Q\Lambda_{k-1}Q^{⊤}Q\Lambda_{k-1}Q^{⊤}{\text{y}}_{k}}{{\text{y}}_{k}^{⊤}Q\Lambda_{k-1}Q^{⊤}{\text{y}}_{k}}=\frac{{\text{z}}_{k}^{⊤}\Lambda_{k-1}^{2}{\text{z}}_{k}}{{\text{z}}_{k}^{⊤}\Lambda_{k-1}{\text{z}}_{k}},$$

with **z**_*k*_=*Q*^⊤^**y**_*k*_. Using the Rayleigh quotient, we obtain

A 7 $$0\leq \lambda_{\min}\leq \frac{{\text{z}}_{k}^{⊤}\Lambda_{k-1}^{2}{\text{z}}_{k}}{{\text{z}}_{k}^{⊤}\Lambda_{k-1}{\text{z}}_{k}}\leq \lambda_{\max}\leq \frac{\mathrm{tr}({\text{y}}_{k}^{⊤}P_{k-1}^{⊤}P_{k-1}y_{k})}{\varsigma_{k}+\delta }=\frac{\varsigma_{k}}{\varsigma_{k}+\delta }\leq 1,$$

with $\lambda_{\min}=\min\{\lambda_{1},\lambda_{2},\ldots ,\lambda_{N}\}$ and $\lambda_{\max}=\max\{\lambda_{1},\lambda_{2},\ldots ,\lambda_{N}\}$. Thus, the convergence time is related to both the output vector **y**_*k*_ and the weight error covariance matrix *P*_*k*−1_.

In practice, we choose the unit matrix *P*_0_=*I*, thus the initial mean square deviation *J*_0_=*tr*(*P*_0_)=*N*. From equation (A.4), we find *J*_1_=*N*−∥**y**_1_∥^2^/(∥**y**_1_∥^2^+*δ*)≈*N*−1. For each recursion step *k* and from equation (A.7), we assume *J*_*k*_≈*N*−*k*. Therefore, the fastest convergence time is about *N* times sampling time Δ*t*, i.e. the recursion step *k*=*N*. The larger the parallel array size *N* is, the longer the convergent time is. We must note that this is an ideal assumption of convergence time *N*Δ*t*, and the experimental results of figure 2 show that the intrinsic time scale for the adaptive algorithm is larger than *N*Δ*t*.

## References

[1] Gammaitoni L 1995 Stochastic resonance in multi-threshold systems. *Phys. Lett. A* 208, 315–322. (doi:10.1103/PhysRevLett.84.2310)

[2] Stocks NG 2000 Suprathreshold stochastic resonance in multilevel threshold systems. *Phys. Rev. Lett.* 84, 2310–2313. (doi:10.1103/PhysRevLett.84.2310) 11018872 10.1103/PhysRevLett.84.2310

[3] Stocks NG 2001 Information transmission in parallel threshold arrays: suprathreshold stochastic resonance. *Phys. Rev. E* 63, 041114 (doi:10.1103/PhysRevE.63.041114) 10.1103/PhysRevE.63.041114 11308826

[4] Stocks NG, Mannella R 2001 Generic noise-enhanced coding in neuronal arrays. *Phys. Rev. E* 64, 030902 (doi:10.1103/PhysRevE.64.030902) 10.1103/PhysRevE.64.030902 11580312

[5] Gammaitoni L, Hänggi P, Jung P, Marchesoni F 1998 Stochastic resonance. *Rev. Mod. Phys.* 70, 223–287. (doi:10.1103/RevModPhys.70.223)

[6] McDonnell MD, Abbott D 2009 What is stochastic resonance? Definitions, misconceptions, debates, and its relevance to biology. *PLoS Comput. Biol.* 5, e1000348 (doi:10.1371/journal.pcbi.1000348) 19562010 10.1371/journal.pcbi.1000348 PMC2660436

[7] Stocks NG, Allingham D, Morse RP 2002 The application of suprathreshold stochastic resonance to cochlear implant coding. *Fluct. Noise Lett.* 2, L169–L181. (doi:10.1142/S0219477502000774)

[8] Oliaei O 2003 Stochastic resonance in sigma-delta modulators. *Electron. Lett.* 39, 1–2. (doi:10.1049/el:20030128)

[9] McDonnell MD, Stocks NG, Pearce CEM, Abbott D 2005 Analog-to-digital conversion using suprathreshold stochastic resonance. In *Proc. SPIE smart structures, devices, and systems II, Sydney, Australia, 13–15 December*, vol. 5649 (ed. SF Al-Sarawi), pp. 75–84. SPIE (doi:10.1117/12.582493).

[10] Nguyen T 2007 Robust data-optimized stochastic analog-to-digital converters. *IEEE Trans. Signal Process.* 55, 2735–2740. (doi:10.1109/TSP.2007.893938)

[11] Zozor S, Amblard PO, Duchêne C 2007 On pooling networks and fluctuation in suboptimal detection framework. *Fluct. Noise Lett.* 7, L39–L60. (doi:10.1142/S0219477507003684)

[12] Hari VN, Anand GV, Premkumar AB, Madhukumar AS 2012 Design and performance analysis of a signal detector based on suprathreshold stochastic resonance. *Signal Process.* 92, 1745–1757. (doi:10.1016/j.sigpro.2012.01.013)

[13] Olson RH, Carr DW 2004 A digital accelerometer array utilizing suprathreshold stochastic resonance for detection of sub-Brownian noise floor accelerations. Sandia National Laboratories, Sandia Report SAND2004-6441.

[14] McDonnell MD, Stocks NG, Pearce CEM, Abbott D 2005 Quantization in the presence of large amplitude threshold noise. *Fluct. Noise Lett.* 5, L457–L468. (doi:10.1142/S0219477505002884)

[15] McDonnell MD, Abbott D 2004 Signal reconstruction via noise through a system of parallel threshold nonlinearities. In *IEEE Int. Conf. Acoustics, Speech, and Signal Processing, Montreal, Canada, 17–21 May*. vol. 2, pp. 809–812. IEEE (doi:10.1109/ICASSP.2004.1326381)

[16] McDonnell MD, Stocks NG, Pearce CEM, Abbott D 2008 *Stochastic resonance: from suprathreshold stochastic resonance to stochastic signal quantization*. Cambridge, UK Cambridge University Press.

[17] Xu L, Vladusich T, Duan F, Gunn LJ, Abbott D, McDonnell MD 2015 Decoding suprathreshold stochastic resonance with optimal weights. *Phys. Lett. A* 379, 2277–2283. (doi:10.1016/j.physleta.2015.05.032)

[18] Xu L, Duan F, Abbott D, McDonnell MD 2016 Optimal weighted suprathreshold stochastic resonance with multigroup saturating sensors. *Phys. A* 457, 348–355. (doi:10.1016/j.physa.2016.03.064)

[19] Hänggi P, Jung P, Zerbe C, Moss F 1993 Can colored noise improve stochastic resonance? *J. Stat. Phys.* 70, 25–47. (doi:10.1007/BF01053952)

[20] Widrow B, Glover JR, McCool JM, Kaunitz J, Williams CS, Hearn RH, Zeidler JR, Dong JRE, Goodlin RC 1975 Adaptive noise cancelling: principles and applications. *Proc. IEEE* 63, 1692–1716. (doi:10.1109/PROC.1975.10036)

[21] Widrow B, Stearns SD 1985 *Adaptive signal processing*. Englewood Cliffs, NJ: Prentice-Hall.

[22] Sayed AH, Kailath T 1994 A state-space approach to adaptive RLS filtering. *IEEE Signal Process. Mag.* 11, 18–60. (doi:10.1109/79.295229)

[23] Douglas SC 1995 Generalized gradient adaptive step sizes for stochastic gradient adaptive filters. In *Int. Conf. Acoustics, Speech, and Signal Processing, 1995* (*ICASSP-95*), *Detroit, MI, 9–12 May*, vol. 2, pp. 1396–1399. IEEE.

[24] Haykin SS 2008 *Adaptive filter theory*. Upper Saddle River, NJ: Pearson Education.

[25] Widrow B, McCool JM, Larimore MG, Johnson CR 1976 Stationary and nonstationary learning characteristics of the LMS adaptive filter. *Proc. IEEE* 64, 1151–1162. (doi:10.1109/PROC.1976.10286)

[26] Widrow B, Walach E 1984 On the statistical efficiency of the LMS algorithm with nonstationary inputs. *IEEE Trans. Info. Theory* 30, 211–221. (doi:10.1109/TIT.1984.1056892)

[27] Simon D 2006 *Optimal state estimation: Kalman, H ∞ and nonlinear approaches*. New York, NY: John Wiley and Sons.

[28] McDonnell MD, Grant AJ, Land I, Vellambi BN, Abbott D, Lever K 2011 Gain from the two-envelope problem via information asymmetry: on the suboptimality of randomized switching. *Proc. R. Soc. A* 467, 2825–2851. (doi:10.1098/rspa.2010.0541)

[29] Mandic DP, Kanna S, Constantinides AG 2015 On the intrinsic relationship between the least mean square and Kalman filters. *IEEE Signal Process. Mag.* 32, 117–122. (doi:10.1109/MSP.2015.2461733)

[30] Zhang L, Song A, He J 2010 Effect of colored noise on logical stochastic resonance in bistable dynamics. *Phys. Rev. E* 82, 051106 (doi:10.1103/PhysRevE.82.051106) 10.1103/PhysRevE.82.051106 21230436

[31] Ma J, Xiao T, Hou Z, Xin H 2008 Coherence resonance induced by colored noise near Hopf bifurcation. *Chaos* 18, 043116 (doi:10.1063/1.3013178) 19123626 10.1063/1.3013178

[32] Bordet M, Morfu S 2013 Experimental and numerical study of noise effects in a Fitzhugh–Nagumo system driven by a biharmonic signal. *Chaos Soliton. Fract.* 54, 82–89. (doi:10.1016/j.chaos.2013.05.020)

[33] Zeng L, Li J, Shi J 2012 M-ary signal detection via a bistable system in the presence of Lévy noise. *Chaos. Soliton. Fract.* 45, 378–382. (doi:10.1016/j.chaos.2011.10.012)

[34] Nozaki D, Mar DJ, Grigg P, Collins JJ 1999 Effects of colored noise on stochastic resonance in sensory neurons. *Phys. Rev. Lett.* 82, 2402–2405. (doi:10.1103/PhysRevLett.82.2402)

[35] Xu B, Li J, Duan F, Zheng J 2003 Effects of colored noise on multi-frequency signal processing via stochastic resonance with tuning system parameters. *Chaos Soliton. Fract.* 16, 93–106. (doi:10.1016/S0960-0779(02)00201-1)

[36] Duan F, Chapeau-Blondeau F, Abbott D 2014 Stochastic resonance with colored noise for neural signal detection. *PLoS ONE* 9, e91345 (doi:10.1371/journal.pone.0091345) 24632853 10.1371/journal.pone.0091345 PMC3954722

[37] Duan F, Chapeau-Blondeau F, Abbott D 2016 Capacity of very noisy communication channels based on Fisher information. *Sci. Rep.* 6, 27946 (doi:10.1038/srep27946) 27306041 10.1038/srep27946 PMC4910081

[38] Collins JJ, Priplata AA, Gravelle DC, Niemi J, Harry J, Lipsitz LA 2003 Noise-enhanced human sensorimotor function. *IEEE Eng. Med. Biol. Mag.* 22, 76–83. (doi:10.1109/MEMB.2003.1195700) 12733463 10.1109/memb.2003.1195700

